# Behavioral dataset for Long-Evans and its schizophrenia-like substrain through several generations

**DOI:** 10.1038/s41597-026-06735-0

**Published:** 2026-02-09

**Authors:** Gábor Kőrösi, Oliver Czimbalmos, Gabriella Kekesi, Gyongyi Horvath

**Affiliations:** 1 Department of Computer Algorithms and Artificial Intelligence, Institute of Informatics, Faculty of Science and Informatics, University of Szeged, Szeged, Hungary; 2 Department of Physiology, Albert Szent-Györgyi Medical School, University of Szeged, Szeged, Hungary; 3 Present Address: Department of Medical Physics and Informatics, University of Szeged, Szeged, Hungary; 4 Present Address: Sztárai Institute, University of Tokaj, Sárospatak, Hungary

**Keywords:** Computational biology and bioinformatics, Computational neuroscience

## Abstract

We present a high-throughput behavioral dataset acquired with Ambitus, an automated reward-based corridor system that records locomotor and exploratory activities and cognitive functions after minimal handling. The collection contains 91 raw and derived variables, each measured across four consecutive trials, for 1,342 Long-Evans rats, including a triple-hit schizophrenia-like substrain (Lisket) bred through 16 generations. All data files, detailed metadata and analysis scripts are openly available on Zenodo. This resource enables longitudinal and multivariate studies of behavioral phenotypes, trans-generational effects, and strain differences, and it provides a benchmark for machine-learning-based marker discovery in rodent models.

## Background & Summary

We present an openly available, comprehensive, multi-generation behavioral dataset for a schizophrenia-like rat model. The dataset comprises 1,342 Long-Evans and Lisket rats tested across 16 generations (2019–2025). Ninety-one parameters describing locomotion, exploration, reward collection and learning were recorded in four sequential trials per animal with the Ambitus corridor system, yielding >360, 000 individual data points. All raw CSV, aggregated Parquet and metadata files, together with analysis notebooks, are available on Zenodo (10.5281/zenodo.16414790)^1^ under a CC-BY 4.0 licence.

Schizophrenia is a complex mental disorder characterized by several symptoms (e.g. positive, negative and cognitive). Modeling this disease using animals is very challenging because of the higher complexity of the human brain compared to the animals. Translational research depends on the relevance of animal models that replicate several signs of a human disorder. Rodents are valuable tools for studying human diseases, although existing animal models cannot fully replicate clinical conditions, especially neuropsychiatric disorders, including schizophrenia. Genetic and environmental factors both contribute to the aetiology of schizophrenia; therefore a multifactorial (“triple-hit”) model is warranted. A “triple-hit” rat substrain, termed Lisket has been developed from the Long-Evans (LE) rats with high behavioral activity and cognition function compared to other strains (e.g. Wistar)^2–4^, by combining post-weaning social isolation, subchronic treatment with the N-methyl-d-aspartate [NMDA] receptor antagonist, ketamine, and selective breeding based on behavioral phenotype manipulations through 16 generations (G0-G15).

Several methods are available to determine behavioral activity and learning ability of rodents during short time periods, including novel object recognition, hole-board and several types of mazes (e.g. Morris Water-maze, and radial mazes with different number of arms)^5–12^. Other, more sophisticated methods, such as tasks with touch screen or lever press require very intensive, long-lasting learning sessions to acquire the tasks^13–17^. To balance throughput and richness, we designed the Ambitus apparatus combining corridor and hole-board elements to record behavior automatically in a rectangular pathway with side-boxes^18^. The Ambitus detects automatically the behavior of animals during simple tasks. We hypothesized that food-deprived rats would collect food rewards from the first trial without prior acclimatization in the corridor system. Sequential trials with paradigm alterations were designed to detect behavioral changes within a single testing session. The instrument can provide rapid and quantitative behavioral readouts within minutes data about the locomotor and exploratory activities and learning capacity of animals within a short time^18^.

While the reliability of Ambitus has been demonstrated in specific paradigms^18^, the present Data Descriptor releases the full seven-year dataset in a FAIR format. By providing comprehensive raw and processed files plus detailed metadata, the resource enables researchers to estimate heritability across generations, identify behavioral markers, validate translational models of schizophrenia, and benchmark machine-learning pipelines. The scale of the dataset further supports pharmacological or toxicological screening, as well as studies of age-related cognitive decline and neural plasticity. Usage examples, step-by-step loading instructions and baseline analysis notebooks accompany the archive to facilitate immediate reuse. Together, these materials furnish an open, extensible platform for behavioral neuroscience and data-driven discovery.

## Methods

### Animals and ethical approval

Long-Evans, and Lisket rats in both sexes during a 7-year period were involved in the study. Animals were treated in accordance with the guidelines set by the Government of Hungary for the humane treatment of animals. All experiments were approved by the Hungarian Ethical Committee for Animal Research (RN: XIV/1248/2018 and XIV/1421/2023) and conducted in compliance with the EU Directive 2010/63/EU and the Animal Research: Reporting of In Vivo Experiments (ARRIVE) guidelines 2.0. Animals were kept with a 12h light/dark cycle under conditions of controlled temperature (22 ± 1^°^C) and before the tests the animals were food deprived for two days, but water was always freely available. The experimental procedures were performed between 8:00 a.m. and 4:00 p.m. in a room with dim general light without external cues.

### Generation protocol and breeding

Based on earlier studies performed with Wistar and its schizophrenia-like substrain (Wisket)^10,18,19^, both LE (control) and Lisket (male and female) rats were weaned at 3 weeks of age. The Lisket rats were housed individually for 28 days and treated with ketamine (30 *mg*/*kg*, 4 *mL*/*kg*) intraperitoneally on 5 consecutive days on the 2nd week of isolation^20^. Subsequently, the animals were rehoused (2-3 animals/cage). LE rats were socially reared with no treatment. The estrus cycle of the female rats was allowed to vary randomly and was not measured or controlled. Three months old control and Lisket rats were selected for mating^10^. To minimize inbreeding sibling mating was avoided. Generally, 6 males were housed with 12 females in sex cages to let one male mate with two females for two weeks, after which the females were reared alone to ensure undisturbed delivery of the offspring. Generation 0 Lisket animals were exposed only to social isolation and ketamine treatment, while the subsequent generations were also selectively bred. In total 1,342 rats of 16 generations (G0-G15) during a 7-year period (year 0-6) were investigated.

### Ambitus apparatus

**Fig. 1:**
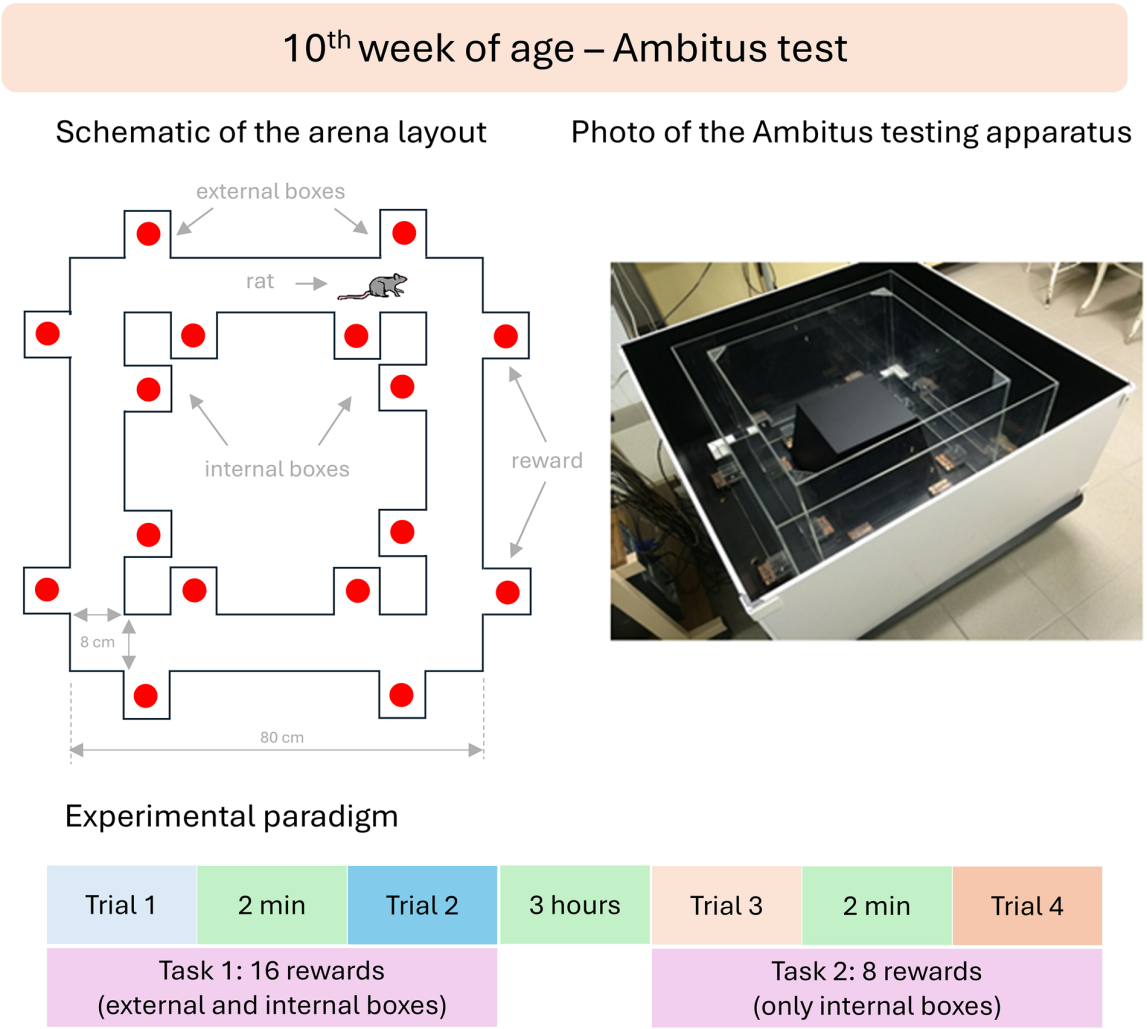
Ambitus apparatus: ground plan of the corridor with 16 side-boxes equipped with photobeams and experimental paradigm.

The Ambitus apparatus, which is designed as a reward-based cognitive test, consists of a rectangular corridor constructed of clear Plexiglas on a black floor with an outer diameter of 80 cm, width of 8cm and height of 50 cm (Fig. 1)^18^. The rats can move around the track between the walls in forward and backward directions. Each corridor contains four side boxes (5 × 5 × 5 cm; 2-2 at the inner and external sides of one corridor, altogether 16) for food rewards (puffed rice, 20*mg*). Each box and the middle of each corridor are fitted with infrared LED (light emitting diodes) at one side and a photocells at the other side to measure nose-poking activity (as exploration) in the boxes, the collection of the rewards, and the locomotor activity, respectively, with 1 ms time resolution, and stores the information about the location of the animals and the duration and the number of visits. Thus, the Ambitus apparatus allowed the analysis of several behavioral parameters, whose definitions and calculation methods are described in detail in the accompanying data descriptor document available in Zenodo^1^. The apparatus was installed in a room where no other activities took place during the testing procedure. Before each trial the experimenter inserted the food rewards into the side boxes. Trials commenced by placing the rats into the same starting point within the corridor (Fig. 1); thereafter, the experimenter immediately left the room. The animals were allowed to explore the corridor and collect food rewards for 5 min (300s; cut-off time). The apparatus was cleaned with 70% alcohol after each rat.

### Behavioral test protocol

At the age of 10 weeks the animals underwent the Ambitus test, as described previously (Fig. 1)^18,21^. Shortly, access to food was restricted 48 h prior to the test, to keep the animals motivated to perform the task. Two types of tasks were employed: Task 1 was performed (Trials 1-2, 2 min apart; all boxes were filled with reward) in the morning, and Task 2 (Trials 3-4, 2 min apart; only the inside boxes were baited) was performed 3 h later. Thus, for all the parameters, 4 values are available, which allow the determination of their trial-dependent changes. For the purposes of the breeding program, selected behavioral variables were categorized using a quartile based scoring scheme adapted from Kékesi *et al*.^19^. Continuous measures including locomotor and exploratory activity, learning capacity and effective exploration were ranked across animals. Values in the lowest quartile were assigned a score of 0, values in the highest quartile a score of 2, and intermediate values a score of 1. The resulting composite scores were used to classify animals into low and high risk categories for a schizophrenia like phenotype.

## Data Records

The dataset is provided in two files: a separated value (csv) and one parquet file:

- **Raw dataset** (trial-level Parquet): This file contains raw trial-level data, where each row corresponds to a single trial for a specific animal. This allows detailed analysis of within-subject behavior over repeated sessions. The structure and variable definitions are also described in ambitus_feature_descriptor.pdf in Zenodo^1^. **File name:** ambitus_0_15_log_04_08_2025.parquet
- **Processed dataset** (ML-ready CSV): This file contains one row per animal, summarizing behavioral performance across four Ambitus trials. It does not include features that are used for calculations and the ones where the feature does not exist. Metadata such as group (LE or Lisket), generation, sex, and testing date are also included. A complete description of all variables is provided in the accompanying data descriptor document ambitus_feature_descriptor.pdf available in the same Zenodo repository^1^. **File name:** ambitus_0_15_ml_ready_04_08_2025.csv

Each animal has a unique identifier and is associated with 4 trials. Feature names are systematically constructed to indicate the source (e.g. trial index) or the applied statistical transformation (e.g., ratio_, _%, prefixes)

**Fig. 2:**
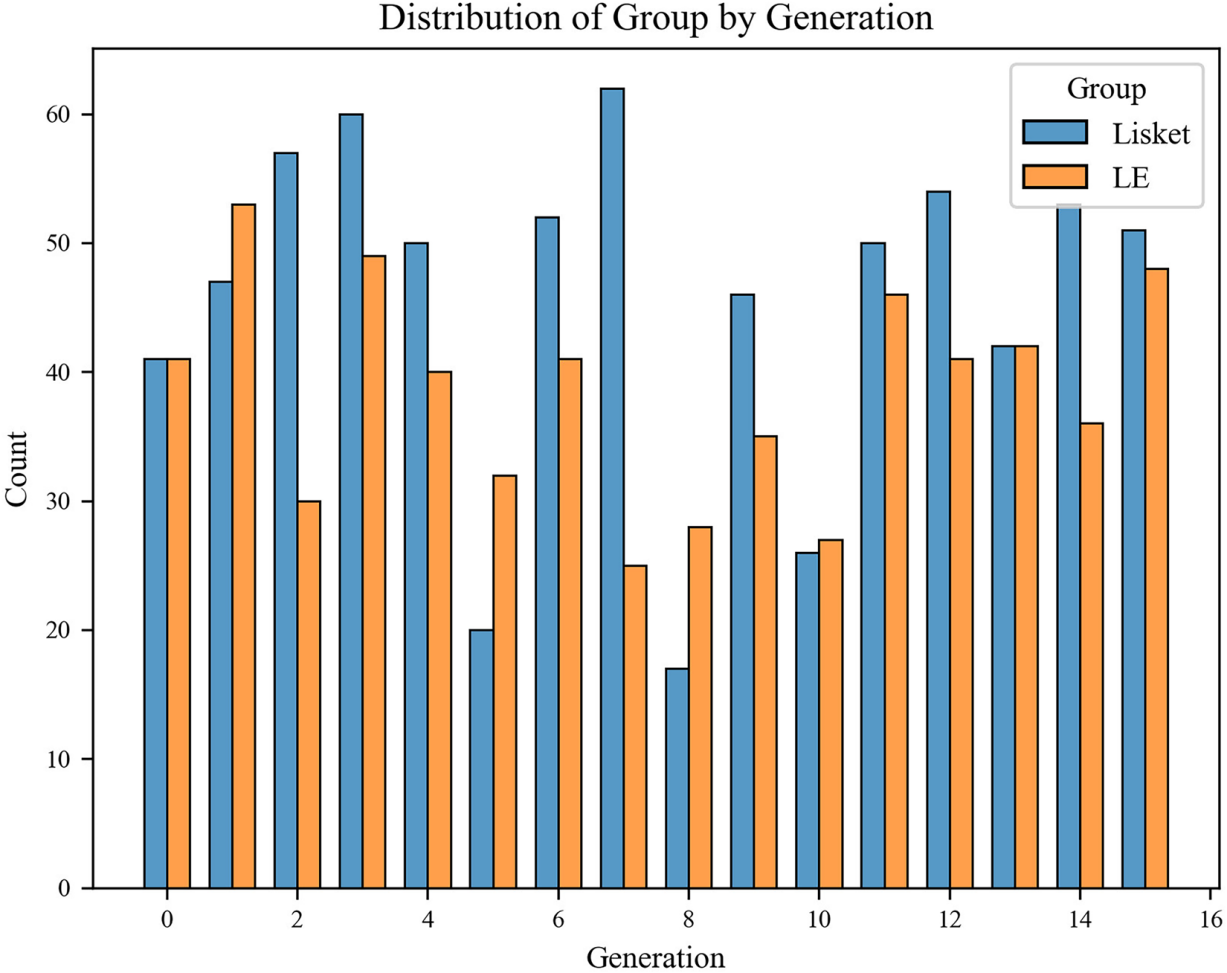
Distribution of animals across experimental groups for each generation. The dataset consists of 728 Lisket (model) and 614 LE (control) rats.

A total of 1,342 animals were recorded, consisting of 728 Lisket and 614 Long-Evans (LE) rats (more details about the distribution see in Fig. 2). The group label distribution is nearly balanced across the dataset, which supports robust comparative analysis. These standardized identifiers facilitate cross-comparison and reproducibility.

All files are released under CC-BY 4.0 and are available at Zenodo (10.5281/zenodo.16414790)^1^. The dataset is deposited on Zenodo as a versioned archive (v6.0, released 28 Oct 2025) containing two sub folders:

- raw/ambitus_0_15_log_04_08_2025.parquet
- processed/ambitus_0_15_ml_ready_04_08_2025.csv

Missing values were treated as NA for the parquet and -1 in the CSV file for more detailed information about the files refer for Table 1.

**Table 1:** Data completeness.

Feature	Percent missing
Expl_E_I_AFT_Calc	58.22
Expl_I_AFT_Calc	58.22
Expl_E_AFT_Calc	58.22
Expl_I_AFT_T_Calc	57.75
Expl_E_AFT_T_Calc	57.75
Expl_E_I_AFT_T_Calc	57.75
Expl_REP_E_AFT_Nr_Calc	57.73
Expl_REP_AFT_Nr_Calc	57.73
Expl_REP_I_AFT_Nr_Calc	57.73
EAT_E_Nr	50.00
EAT_E_%	50.00
Eff_Expl_E	50.00
L_C_E	50.00
EAT_E_T	50.00
Separation	0.60
Expl_E_BEF_Ratio	0.41
Expl_E_TOT_Ratio	0.41
Circle_Nr	0.41
Dir_Change_Nr	0.41
A_E	0.41
Expl_EI_BEF_Loco_ratio	0.34
Expl_E_I_TOT_Loco_ratio	0.34
Expl_E_BEF_Loco_ratio	0.34
Expl_I_BEF_Loco_ratio	0.34
Expl_E_I_BEF_T	0.30
Expl_I_BEF_T	0.30
Expl_E_BEF_T	0.30
Expl_I_TOT_Loco_ratio	0.30
Expl_E_TOT_Loco_ratio	0.30
Expl_E_AFT_Nr	0.28
Expl_I_AFT_Nr	0.28
Expl_E_AFT_T	0.19
Expl_I_AFT_T	0.19
Expl_E_I_AFT_T	0.19
Expl_I_BEF_Calc	0.02

The feature naming convention is followed as Task_Location_Measure (e.g. EAT_E_Nr, number of the collected rewards from the external boxes) the csv file uses the same convention extended with the “_Trial” (e.g. EAT_E_Nr_4. number of the collected rewards from the external boxes at the 4th trial).

## Technical Validation

All correlations were computed using Spearman rank correlation (two-tailed, Benjamini-Hochberg (BH)-adjusted). This non-parametric method was selected due to the non-normal distribution of several behavioral variables and the anticipated monotonic, potentially non-linear relationships among them. Spearman correlation provides more robust and reliable estimates in the presence of skewed distributions and outliers, which are common in behavioral datasets. All analyses and figures reported in this section can be fully reproduced with the notebook *technical*_*validation*. *ipynb* (GitHub tag vol 6.1, Zenodo 10.5281/zenodo.16414790)^1^.

## Data completeness

We quantified the amount and structure of missing data in the processed table that contains one row per animal (1,342 rows × 91 features; trial-wise variables are aggregated). For each variable we calculated the proportion of missing entries (pandas 2.3, pair-wise deletion) and summarised the results in Table 1. Missing values primarily arise from low behavioural activity, which resulted in incomplete reward collection and consequently the absence of “after” measurements. This effect was more pronounced in the Lisket strain, leading to a higher frequency of “non-visit” trials. The prevalence of such trials represents an informative behavioural characteristic that can be quantified and analysed in downstream studies.

**Fig. 3:**
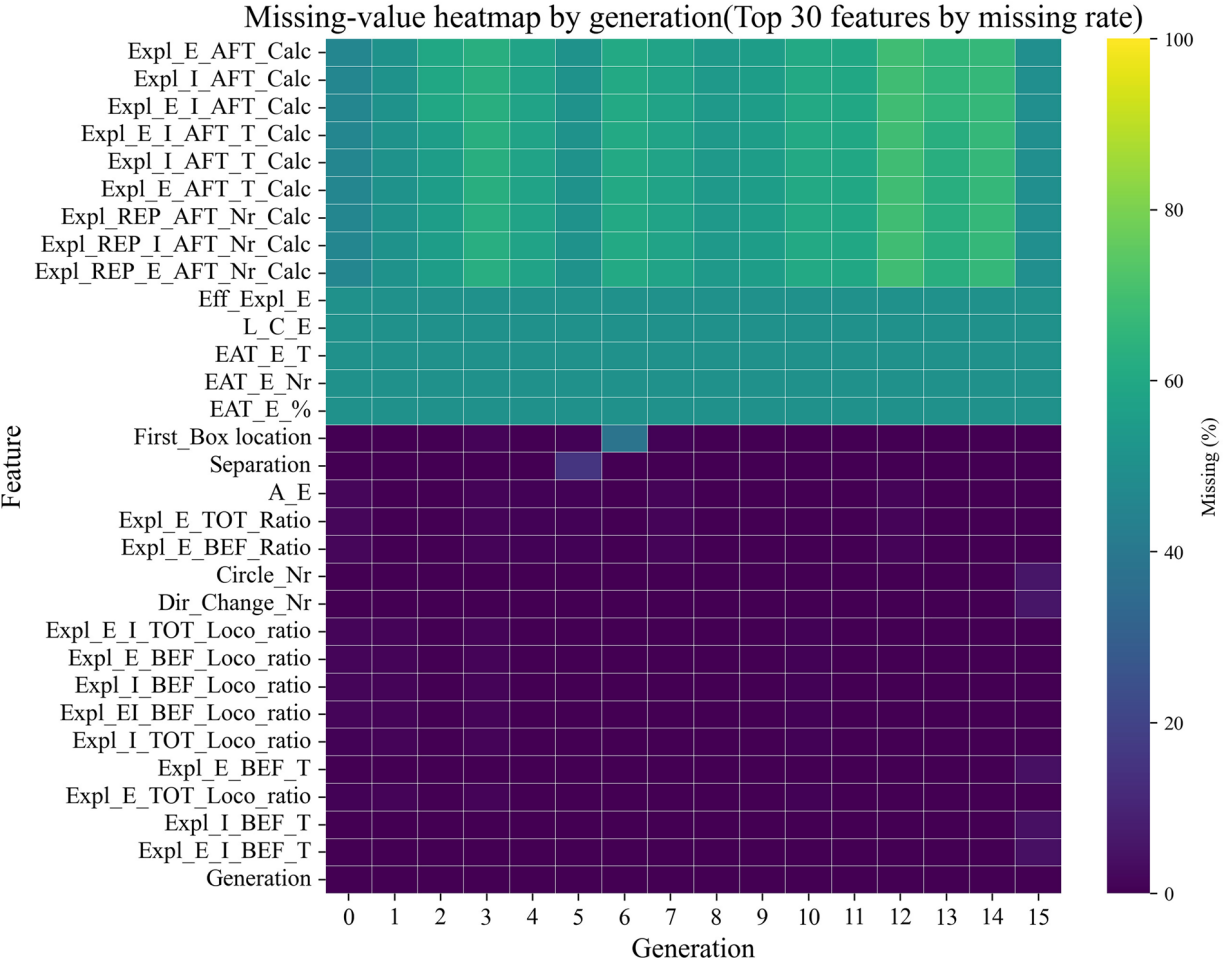
Missing-value heat-map by generation. Percent missing values (0–60%) for the 30 most affected features are displayed for generations 0 to 15. Colours range from dark purple (0%) to yellow (60%). Underlying code is available in the GitHub repository (vol 6.1).
- **Generation consistency:** A heat-map of the 30 most affected features (Fig. 3) indicates that the missing-value pattern is remarkably uniform across the 16 generations: the small rise seen in G14-G15 mirrors the slightly shorter average trial length in these cohorts, not a sensor outage. This supports the assumption that no generation-specific dropout or batch effect is present.
- **Encoding and handling:** In the released Parquet table missing entries are stored as NaN; in the legacy CSV logs the same positions are coded as −1 and converted to NaN during preprocessing. Users may safely apply list-wise or multiple imputation without violating data-integrity assumptions; an example SimpleImputer workflow is provided in the GitHub notebook *technical*_*validation*. *ipynb* (tag vol 6.1)^1^.

- **Overall coverage:** Eighty-three of the 91 variables show <1% missing values; 55 of these are fully complete. The remaining eight variables belong to the Exploratory-after-task latency group (Expl_E/I_AFT_Calc derivatives) and exhibit the highest loss (*maximum* ≈ 60%; Fig. 3). These metrics are absent whenever an animal failed to visit any side-box during the post-task interval—an expected, protocol-dependent limitation rather than technical failure.

Together, these analyses confirm that the dataset is >99% complete for the vast majority of variables and that the limited missingness is protocol-driven, well documented, and evenly distributed—satisfying the FAIR “quality and reusability” criterion.

Missing values in the legacy parquet file (raw/ambitus_0_15_log_04_08_2025.parquet) arise only when a parameter is undefined for a given task variant (e.g., latency-to-internal-box metrics in Task 2) or when an entire trial failed. In the ML-ready CSV table these cells are encoded as -1 and automatically converted to NaN during preprocessing. Because 83 of 91 features show <1% missingness and the remaining gaps are protocol-dependent (Table 1, Fig. 2), standard list-wise deletion or multiple imputation can be applied without biasing downstream analyses.

## Feature Correlation Analysis

**Fig. 4:**
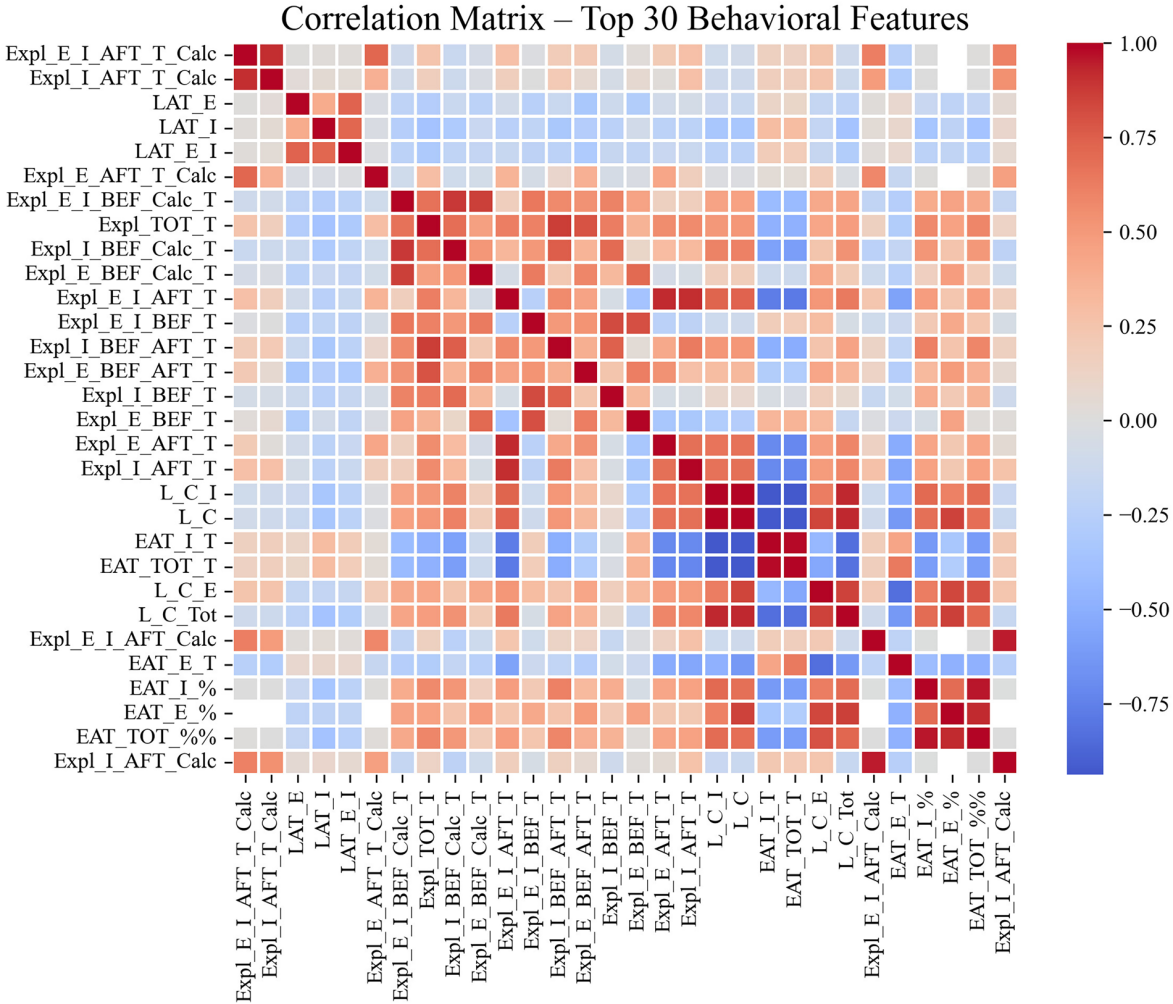
Correlation matrix of 30 behavioral features - Spearman correlation coefficients were calculated between the 30 most variable behavioral parameters derived from the Ambitus testing system. These features were selected based on highest standard deviation across all animals, and represent diverse cognitive, locomotor, and reward-related domains. Strong positive and negative correlations (∣r∣ > 0.7) indicate partially redundant measures or functionally related processes, such as coordinated reward retrieval and exploratory strategies. Underlying code is available in the GitHub repository (tag vol 6.1).

To evaluate internal consistency and redundancy among the 91 behavioral metrics, we calculated pairwise Spearman rank correlation coefficients (pairwise deletion, *n* = 1, 342) using pandas 2.3 and visualised the 30 most variable features (selected by standard deviation) as a heat-map (Fig. 4; notebook *technical*_*validation*. *ipynb* code in GitHub tag vol 6.1). The calculation does not distinguish between trials.

The resulting heatmap (Fig. 4) revealed several interpretable correlation clusters:

- Features derived from similar behavioral constructs (e.g., total exploration time and box visit counts) showed strong positive correlations, indicating potential redundancy or shared underlying processes.
- Measures associated with learning capacity and efficient task performance (e.g., reward collection efficiency, latency to first reward) formed distinct but moderately correlated groups.
- Some parameters (e.g., ratios or normalized variables) exhibited reduced correlation with absolute raw metrics, reflecting their design to capture relative performance patterns.

This analysis highlights both the internal consistency of feature groups and the presence of partially overlapping information, supporting the rationale for dimensionality reduction or feature selection in downstream modeling tasks.

## Generational drift

To visualise the absence of systematic cohort effects on well-populated variables, we replaced the two latency metrics that carry the highest proportion of missing values with three representative features that (i) rank among the 20 strongest raw *ρ* values yet (ii) are >98% complete:

- Expl_E_AFT_T - elapsed time (sec) spent in exploratory behavior after task
- Expl_E_AFT_Nr - count of exploratory events after task
- Expl_E_TOT_Loco_ratio - ratio of exploration to locomotion during the entire session

**Fig. 5:**
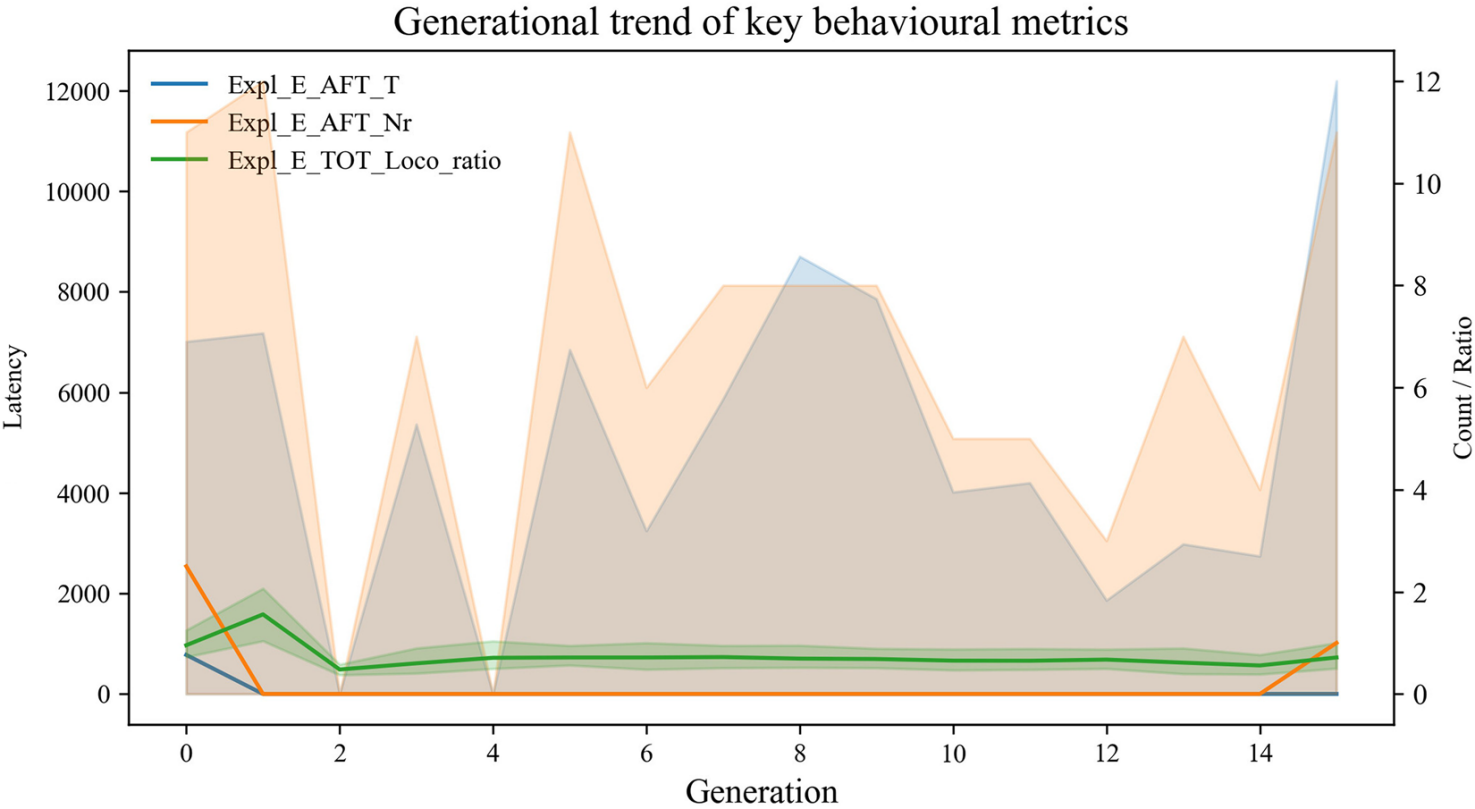
Generational trend of three representative exploratory metrics (median  ± IQR). Lines show the generation-wise median; shaded areas indicate the inter-quartile range. All three variables - Expl_E_AFT_T (blue), Expl_E_AFT_Nr (orange) and Expl_E_TOT_Loco_ratio (green) - display small, non-systematic fluctuations, supporting the conclusion of negligible generational drift. (latency = ms, count = events, ratio = unitless). Underlying code is available in the GitHub repository (tag vol 6.1).

**Fig. 6:**
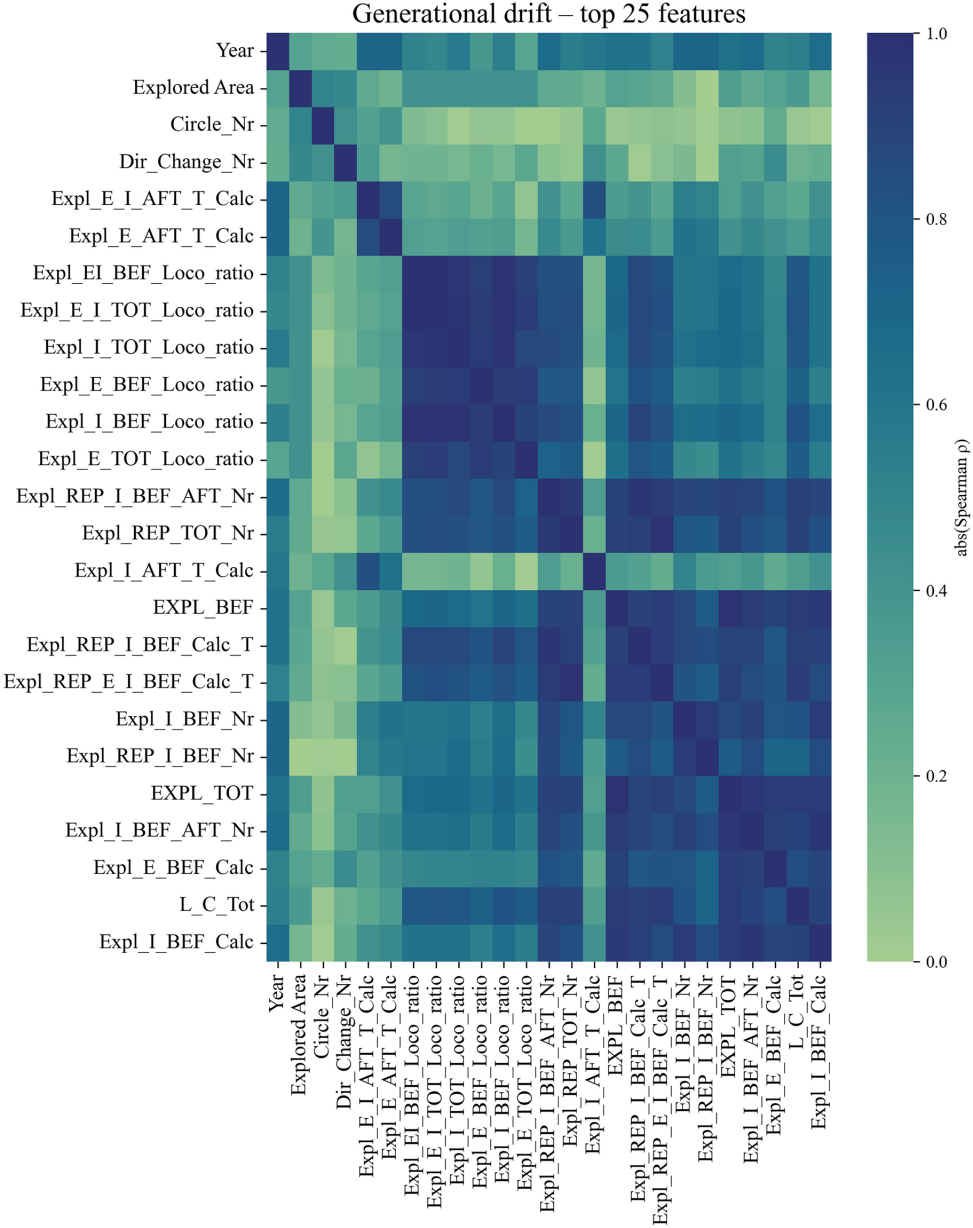
Generational drift heat-map (top-25 features). Absolute Spearman correlations (0-0.25) between generation number and the 25 variables with the strongest drift. The muted colour range and lack of block structure indicate minimal lineage-specific change. Underlying code is available in the GitHub repository (tag vol 6.1).

Median  ± IQR curves for these metrics (Fig. 5) remain essentially flat across the 16 generations, with overlapping inter-quartile bands and no monotonic trend, mirroring the low absolute Spearman coefficients observed. Together with the global heat-map (Fig. 6) this confirms that any residual drift is minimal, protocol-related and unlikely to bias downstream analyses.

Together these findings indicate that the behavioral phenotypes are qualitatively conserved over the seven-year, 16-generation span, satisfying the dataset’s internal-consistency requirement and supporting its reuse for downstream modelling.

## Within-task concordance

Because Trials 1–2 (Task 1) and Trials 3–4 (Task 2) were separated by three hours and differed in bait layout, we evaluated reproducibility with non-parametric Spearman rank correlations coefficients rather than intraclass correlation coefficient (ICC). Median *ρ* was 0.44 for Task 1 and 0.40 for Task 2 (IQR 0.32–0.59 and 0.29–0.51, respectively; Table 2). Locomotor activity and reward-collection counts displayed high concordance (*ρ* ⩾ 0.80), confirming sensor stability, whereas exploration-latency metrics showed low agreement (*ρ* ⩽ 0.25), consistent with rapid habituation and satiety rather than measurement error. The accompanying notebook *technical*_*validation*. *ipynb* (GitHub tag vol 6.1)^1^ reproduces the analysis.

**Table 2:** Task-level Spearman summary.

Task	median_rho	IQR_rho	n_vars	n_reliable
Task1	0.44	0.32–0.59	91	17
Task2	0.40	0.29–0.51	86	8

## Usage notes

This dataset is suitable for supervised and unsupervised behavioral classification, longitudinal modeling of phenotypic drift, and generational tracking of cognitive function. It should be considered that cognitive functions are influenced by several factors, including attention, motivation, and locomotor activity; thus, the learning process cannot be measured in isolation. However, further correlation analyses between the parameters of behavioral activities and cognitive functions may reveal their associations. Users should be aware that certain behavioral metrics may be sensitive to environmental variables such as handling and lighting, particularly in early generations. Trials were conducted under standardized protocols, but raw trial-level data should be normalized before direct comparison across animals. All accompanying Jupyter notebooks (GitHub tag vol 6.1, Zenodo 10.5281/zenodo.16414790)^1^ include clearly marked “playground” cells, allowing users to tweak preprocessing steps, model hyper-parameters and validation schemes.

## Data Availability

All raw behavioural logs, aggregated Parquet/CSV tables, metadata files and folder structure described in this Data Descriptor are openly available on Zenodo under the accession 10.5281/zenodo.16414790^1^. The dataset is released under a CC-BY 4.0 licence. All files required to reproduce the figures, analyses and supplementary materials can be accessed and downloaded without restriction.

## Code availability

All Python scripts and Jupyter notebooks used for feature extraction, preprocessing and illustrative analyses are archived at GitHub (https://github.com/szegedai/ambitus-rat-behaviour-dataset, release vol 6.1) and Zenodo (10.5281/zenodo.16414790)^1^. The repository is distributed under the MIT licence and includes a requirements.txt file to facilitate full reproducibility.
